# Psychological problems in gastroenterology outpatients: A South Australian experience. Psychological co-morbidity in IBD, IBS and hepatitis C

**DOI:** 10.1186/1745-0179-4-15

**Published:** 2008-05-23

**Authors:** Antonina A Mikocka-Walus, Deborah A Turnbull, Jane M Andrews, Nicole T Moulding, Ian G Wilson, Hugh AJ Harley, David J Hetzel, Gerald J Holtmann

**Affiliations:** 1 Department of Epidemiology & Preventive Medicine, Monash University, The Alfred, Level 3, Burnet Tower, 89 Commercial Rd, Melbourne 3004, VIC, Australia; 2 School of Psychology, University of Adelaide, Level 4, Hughes Building, Adelaide 5005, SA, Australia; 3 Department of Gastroenterology and Hepatology, Royal Adelaide Hospital, North Wing Q7, Adelaide 5005, SA, Australia; 4 School of Social Work and Social Policy, University of South Australia, Magill Campus, H1-32, Magill 5068, SA, Australia; 5 School of Medicine, University of Western Sydney, Locked Bag 1797, Penrith South DC NSW 1797, Australia

## Abstract

**Background:**

In independent studies, IBD, IBS and HCV have each been associated with a substantially increased risk of psychological problems such as depression and anxiety and impairment of quality of life compared to the general healthy population. However, the relative psychological burden for each of these diagnoses is unknown as it has never been compared contemporaneously at one institution. Current local data are therefore needed to enable an evidence-based allocation of limited clinical psychological resources.

**Methods:**

Overall, 139 outpatients (64 IBD, 41 HCV, and 34 IBS) were enrolled in this cross-sectional study. The HADS, SCL90, SF-12 and appropriate disease-specific activity measures were administered. Differences between groups were assesed with ANOVA, the Chi-Square test and the independent samples t-test (two-tailed).

**Results:**

Each of the three groups had significantly lower quality of life than the general population (p < 0.05). Overall, a total of 58 (42%) participants met HADS screening criteria for anxiety and 26 (19%) participants for depression. The HCV group had a significantly higher prevalence of depression than either of the other groups (HCV = 34%, IBS = 15% and IBD = 11%, p = 0.009). In the SCL90, the three disease groups differed on 7 out of 12 subscales. On each of these subscales, the HCV group were most severely affected and differed most from the general population.

**Conclusion:**

Patients with these common chronic gastrointestinal diseases have significant impairment of quality of life. Anxiety is a greater problem than depression, although patients with HCV in particular, should be regularly monitored and treated for co-morbid depression. Evaluation of specific psychological interventions targeting anxiety is warranted.

## Background

Psychological co-morbidities are prevalent in patients with chronic gastrointestinal and hepatologic disorders [1]. However, securing clinical funding to target these problems in a systematic fashion is difficult even though psychological interventions may improve patients' outcomes [2] and reduce health care utilisation [3]. There are multiple conflicting demands for resources in clinical gastroenterology and traditionally each subspecialty area within a unit has sought "special case" status. Therefore, direct comparisons of psychological co-morbidity between these common gastrological conditions within a unit have not been previously performed. To fill this evidence gap we thus sought to directly compare the prevalence of depression and anxiety disorders in three common chronic gastroenterological disorders to provide most up-to-date local data which enable more efficient, evidence-based use of resources.

Inflammatory bowel disease (IBD), irritable bowel syndrome (IBS) and chronic hepatitis C (HCV) are chronic diseases frequently encountered in gastroenterology. Even though the conditions differ in their aetiology and symptoms, they all have been associated with an increased prevalence of psychological co-morbidities in independent studies [4-6]. Despite studies of the co-occurrence of IBS in IBD [7] as well IBD and HCV [8], there is little research which directly compares quality of life and the prevalence of psychological disorders between these three diseases. Interestingly, common treatments for two of these conditions are associated with mood disorders. This is particularly so for interferon-alpha treatment in patients with hepatitis C and corticosteroids in IBD. Thus, these patients' depression and anxiety may in part be iatrogenic. On the other hand, IBD and IBS commonly coexist and although, traditionally, IBD is regarded as an inflammatory disorder and IBS as a functional disorder, recently, it has become increasingly difficult to make absolute distinctions between the two conditions, as current research now provides evidence on the existence of inflammatory abnormalities in the gut of patients with IBS [9,10]. Somewhat puzzlingly, IBS, which causes the least objectively quantifiable disturbance to gastrointestinal function, has been thought to have the highest prevalence of anxiety and depression among these three conditions; however, these data come from non-comparative studies [6,11]. Thus, the current study aims to determine whether real differences exist among IBD, IBS and HCV with respect to depression, anxiety and quality of life by comparing patients recruited contemporaneously from a single site.

## Methods

### Participants and recruitment

Patients with clinically diagnosed (by gastroenterologists) IBD, IBS and HCV were recruited to this study between November 2005 and June 2006 through the Outpatient Clinic at the Department of Gastroenterology and Hepatology at the Royal Adelaide Hospital. Participants must have had sufficient knowledge of English to understand and answer the questionnaires. HCV patients with known cirrhosis of the liver were excluded from the study. The study was described to potential participants and each participant was provided with a consent form, questionnaires and a reply paid envelope. Participants were asked to complete a survey comprising a measure of anxiety and depression; a measure of a broad psychological profile; a measure of quality of life; and a measure of disease activity (in the HCV group, a blood test indicating active viral replication was taken as a dichotomous variable for active versus inactive disease).

### Measurements

#### Anxiety and depression screening

Screening for anxiety and depression was conducted with the Hospital Anxiety and Depression Scale (HADS). The HADS contains 14 questions graded on a 4-point Likert scale (0–3), with subscales of anxiety (seven items) and depression (seven items), with a sum score ranging from 0 to 21 for each of anxiety and depression. A cut-off value for clinical caseness is 7. Scores between 8 and 10 are interpreted as possible cases, and = 11 as certain cases. Importantly, the HADS is a screening measure and can only be used to estimate a likely prevalence of anxiety and depression, and not to establish a firm diagnosis. The latter can only be made by a psychologist or a psychiatrist using a structured interview based on the DSM-IV [12].

#### Psychological profiles

Participants' broad psychological profiles were assessed by the SCL-90-R Symptom Checklist-90-R (SCL-90). This is a 90-item self-report instrument. The SCL-90 contains 9 subscales: Somatization, Obsessive-Compulsive, Depression, Anxiety, Hostility, Phobic Anxiety, Paranoid Ideation, and Psychoticism. It also comprises three global indices: Global Severity Index (GSI), Positive Symptom Distress Index, and Positive Symptom Total [13]. There are no specified cut-off values for this scale; however, caseness can be identified when the GSI score ≥ 63 (after the transformation into the T score) or when any two primary dimension scores are ≥ 63.

#### Quality of life

Screening for quality of life was performed with the Short Form 12 Health Survey (SF-12). The SF-12 contains two subscales: the Mental Component Summary (MCS) and the Physical Component Summary (PCS) [14]. Scores for each subscale range between 0 and 100, with increasing values indicating better health.

#### Disease activity

Disease activity in IBD participants was assessed with the Crohn's Disease Activity Index (CDAI) [15] or the Simple Clinical Colitis Activity Index (SCCAI) [16] as appropriate. A CDAI score ≤ 150 was considered remission in CD and a SCCAI score of ≤ 2 was considered remission in UC. The CDAI was measured via retrospective assessment of symptoms.

Disease activity in IBS patients was measured by two questions added into the general health survey: "Have you got satisfactory control of your IBS symptoms over the last 3 months?" and "Are you now feeling better or worse when compared to your last visit in the clinic?". Answering "yes" and "better" to these questions was considered remission in IBS.

Disease activity in HCV (or, more precisely, ongoing viral replication) was measured by RT-PCR HCVRNA. RT-PCR HCVRNA "Not detected" was considered remission of HCV. RT-PCR HCVRNA is a marker of an ongoing infection. Values for the presence of HCV RNA in the HCV patients and heamatocrit in the IBD patients were routinely conducted by the treating physicians as per the usual treatment schedule.

### Ethical considerations

The study was approved by the Royal Adelaide Hospital Research Ethics Committee. Participants were aware that their care did not in any way depend on participation or non-participation in the study. Each participant gave written informed consent. The work was performed in accordance with the principles of the 1983 Declaration of Helsinki [17].

### Statistical analysis

Normality was assessed with the use of qq-plots. Differences between groups (when the distribution was normal) were assesed with a one way analysis of variance (ANOVA). Results were adjusted for the multiple comparisons with Tukey's HSD adjustment. Categorical data were analysed using contingency tables with the Chi-Square test. A five-step hierarchical regression of baseline group differences in terms of demographic characteristics and mean scores for the three main scales was conducted. At first, the mean effects of disease, sex, age, education, activity and years since diagnosis were entered. In the second step, the disease interactions with sex and age were added. In the third step, the disease interactions with years since diagnosis were entered. The fourth step involved adding disease interactions with disease activity, and in the final step, the disease and level of education interactions were entered. At each step the "r^2^" and the "r^2^" change were reported. A change in the "r^2^" was tested statistically. Differences between groups and the general population for quality of life were calculated with the independent samples t-test (two-tailed).

## Results

Of 437 patients approached to participate in the study, 139 accepted the invitation (31.8% of all patients invited). Of these, 64 patients had been previously diagnosed with IBD: 33 with UC and 31 with CD. Forty one patients had been previously diagnosed with HCV and 34 with IBS. Twenty six percent of all invited patients with IBD, 43.6% of all invited patients with HCV, and 32.4% of all invited patients with IBS accepted the invitation to participate in the study. Respondents and non-respondents did not differ with respect to gender and public/private health services user status. No other demographic information was available for non-respondents as the hospital ethics committee did not allow the authors to collect any other data if a patient did not consent to participate in the study.

The range of CDAI scores was between 0 and 319 and the SCCAI range was 0 to 10. As regards treatments that may influence psychological well being; six IBS patients were on antidepressants. Ten HCV patients were receiving combination therapy with interferon & ribavirin and seven were taking antidepressants. Only one HCV patient was taking both a combination therapy and an antidepressant. In the IBD group, one CD patient and eight UC patients were on antidepressants while three CD patients and four UC were on oral prednisolone.

### Demographic characteristics of participants with HCV, IBD and IBS

Overall, 61 percent of participants were female (See Table 1). The proportion of female to male participants in the IBS group was significantly higher than in the IBD or the HCV group (χ^2 ^(2) = 6, p = 0.050). Participants did not significantly differ in their level of education (χ^2^(6) = 5.48, p = 0.483).

**Table 1:** Gender and education in HCV, IBD and IBS participants

Disease		HCV (n = 41)	IBD (n = 64)	IBS (n = 34)	Total (n = 139)
Number of participants/percent		N (%)	N (%)	N (%)	N (%)
Gender*	Male	21 (51)	25 (39)	8 (23)	54 (39)
	Female	20 (49)	39 (61)	26 (77)	85 (61)
Education	Primary	3 (7)	3 (5)	3 (9)	9 (7)
	Secondary	22 (54)	29 (45)	13 (38)	64 (46)
	Trade/TAFE	1 (2)	8 (12)	5 (15)	14 (10)
	Tertiary	11 (27)	18 (28)	12 (35)	41 (29)
	Not specified	4 (10)	6 (10)	1 (3)	11 (8)

* p ≤ 0.05

The HCV group was significantly younger than the IBS group and had significantly shorter period since the diagnosis than the IBD group (p ≤ 0.05) (See Table 2) whilst the number of years with symptoms was similar in all the three groups (p > 0.05).

**Table 2:** Age, years since diagnosis, years with symptoms in HCV, IBD and IBS participants

	HCV Mean (SD)	IBD Mean (SD)	IBS Mean (SD)	F	p
Age	45(10)*	51 (15)	54 (13)*	3.793	0.023
Years since diagnosis	7 (4)*	14 (10)*	10 (9)	6.808	0.001
Years with symptoms	16 (9)	16 (11)	16 (13)	0.008	0.992

* differences significant at the level of p < 0.05

Groups did not significantly differ in their disease activity (Table 3).

**Table 3:** Disease activity in HCV, IBD and IBS

Disease	HCV (n = 41)	IBD (n = 64)	IBS (n = 34)	Total (n = 139)
Number of participants (%)	N (%)	N (%)	N (%)	N (%)
Active disease	23 (56)^1^	23 (36)	18 (53)	64 (46)
Not active disease	18 (44)	41 (64)	14 (41)	73 (52)
Not specified	0 (0)	0 (0)	2 (6)	2 (2)

^1 ^Active viral replication was used as a measure of disease activity in HCV

Hierarchical regression showed significant demographic differences on two scales: the SF-12 Physical and the SCL90 PST (data not presented). Older participants tended to have poorer physical quality of life than younger participants (p = 0.014); those with HCV tended to have poorer physical quality of life than those with UC (p = 0.012); and those participants who had disease for a longer time had better physical quality of life than those reporting a briefer duration of disease (p = 0.016). Moreover, participants with CD tended to have poorer physical quality of life than UC participants (p = 0.038), but their physical quality of life increased with age (p = 0.037). HCV participants with longer disease had poorer physical quality of life (p = 0.050). Additionally, HCV participants with the longer disease had higher (poorer) PST score than those with the shorter disease (p = 0.032).

### Differences in psychological profiles and quality of life between HCV, IBD and IBS participants

Groups did not significantly differ in mean scores for anxiety or depression as measured by the HADS nor in either physical or mental components of quality of life (p > 0.05) (Table 4). Groups did not significantly differ on the following subscales of the SCL90: the Interpersonal-Sensitivity subscale, the Depression subscale, the Hostility subscale, the Psychoticism subscale and the PSDI subscale, either (p > 0.05).

**Table 4:** Group differences on the HADS, SF-12 and SCL90 in HCV, IBD and IBS participants by one-way ANOVA

	HCV Mean (SD)	IBD Mean (SD)	IBS Mean (SD)	F	p
HADS Anxiety	6.97 (4.83)	6.57 (3.44)	7.97 (2.92)	1.498	0.227
HADS Depression	5.36 (4.96)	4.07 (2.86)	4.29 (3.70)	1.513	0.224
SF12 Mental	44.59 (13.41)	49.10 (10.76)	46.36 (10.09)	2.024	0.136
SF12 Physical	43.41 (11.66)	45.93 (10.85)	43.11 (11.30)	0.974	0.380
Somatization	61.17 (12.90)*	55.35 (10.18)*	59.85 (8.23)	4.277	0.016
Obsessive-Compulsive	61.43 (11.59)*	56.48 (8.17)*	59.05 (9.77)	3.331	0.039
Interpersonal Sensitivity	60.43 (11.96)	55.43 (11.03)	57.52 (11.48)	2.397	0.095
Depression	62.26 (12.50)	58.04 (9.94)	59.99 (10.16)	1.909	0.152
Anxiety	57.63 (13.61)*	51.79 (9.74)*	56.82 (8.77)	4.462	0.013
Hostility	57.43 (11.98)	52.96 (9.86)	55.85 (11.00)	2.281	0.106
Phobic Anxiety	54.95 (11.60)*	51.54 (8.28)	49.79 (7.96)*	3.073	0.050
Paranoid Ideation	56.58 (11.99)*	50.73 (10.18)*	51.88 (11.86)	3.568	0.031
Psychoticism	58.24 (10.58)	54.46 (8.97)	56.23 (10.18)	1.878	0.157
GSI	62.00 (12.22)*	55.95 (9.66)*	59.94 (9.75)	4.450	0.013
PST	60.92 (11.34)*	55.93 (9.51)*	58.85 (9.33)	3.201	0.044
PSDI	57.97 (9.78)	54.29 (9.80)	56.61 (8.36)	1.997	0.140

* differences significant at the level of p < 0.05

The groups did, however, differ on the remaining 7 of 12 SCL90 subscales (Somatization, Obsessive-Compulsive, Anxiety, Phobic Anxiety, Paranoid Ideation, GSI and PST). The HCV group had the highest mean score on each of these subscales of the SCL90, indicating higher psychological morbidity in this group compared to both the IBS and IBD groups. In particular, for the SCL90 Somatization, Obsessive-Compulsive, Anxiety, Paranoid Ideation, GSI and PST analysis, the HCV group had a higher score than the IBD group (p = 0.05). For the SCL90 Phobic Anxiety analysis, the HCV group had a higher score than the IBS group (p = 0.048).

When we conducted an additional analysis (data not shown) and compared the means on all the scales between sexes in the whole group of patients, we observed interestingly that male participants had higher scores on the SCL90 Somatisation subscale (p = 0.021), the SCL90 Depression subscale (p = 0.042) and the SCL90 PST subscale (p = 0.039) than female participants, indicating higher levels of psychological co-morbidity in male as compared to female participants.

### Prevalence of anxiety and depression

Overall, 58 participants (42%) fulfilled anxiety criterion for caseness (score > 7) and 26 participants (19%) fulfilled depression criterion for caseness (score > 7) (See Table 5). There was no significant difference between groups in the prevalence of anxiety (χ^2 ^(2) = 0.948, p = 0.623). However, there was a statistically significant difference in the prevalence of depression between the groups (HCV = 34%, IBS = 15% and IBD = 11%, p = 0.009), with the HCV group having significantly higher prevalence of depression than the IBD and IBS groups.

**Table 5:** Percentage of anxious and depressed patients in three disease groups based on the HADS criteria for caseness (score > 7)

Disease	HCV (n = 41)	IBD (n = 64)	IBS (n = 34)	Total (n = 139)
Number of participants/percent	N (%)	N (%)	N (%)	N (%)
Anxiety	18 (44)	24 (37)	16 (47)	58 (42)
Depression	14 (34)*	7 (11)*	5 (15)*	26 (19)

*χ^2 ^(2) = 9.326, p = 0.009

### Quality of life comparisons between IBD, HCV and IBS groups and the general population

As the rate of psychological disorders in the three groups was not high, comparisons with the normal population in terms of quality of life were conducted to determine whether, and by how much, the three disease groups differed from the healhy population.

**Figure 1:**
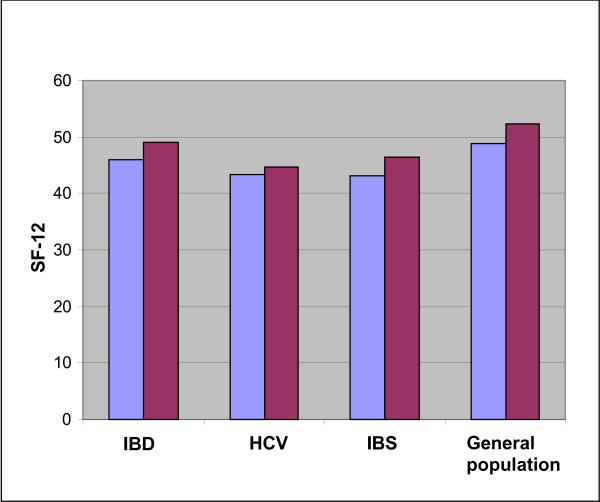
**Quality of life in IBD, HCV, IBS and general population**.  Physical quality of life.  Mental quality of life.

Significant differences in both physical and mental components of quality of life were detected between each disease group and the general South Australian population (Figure 1). The IBD group, the HCV group and the IBS group each had poorer physical quality of life (means of 45.93, 43.41, and 43.11, respectively vs. 48.73; p = 0.032, p = 0.003 & p = 0.004 respectively) and poorer mental quality of life (means of 49.10, 44.59, and 46.36, respectively vs. 52.3; p = 0.009, p = 0.000 & p = 0.000 respectively) than the general South Australian population.

## Discussion

This study is novel in that it compares psychosocial co-morbidities in contemporaneous samples of patients with IBD, IBS and HCV in Australia. However, it is important to acknowledge that this study results need to be interpreted with caution due to the low participation rate of only 32%. Unfortunately, due to the lack of full data on non-participants, we are unable to speculate on the magnitude of any bias and in which direction this bias may fall. Whilst one may hypothesize that patients with more severe psychological disease were less likely to participate, it could equally be the case that less affected patients thought the study of less relevance to them and declined. Realistically, the low participation rate could only be improved by incorporating these screening measures into the routine clinical care of these patients, as there would then be no issues of consent.

Whilst previous studies have reported a high prevalence of psychological disorders in IBS [18] and only a moderate level of these disorders in HCV [19], these data come from different units at different time points. However, when these patients are compared contemporaneously in the one clinic, the current study clearly showed that patients with HCV were the most depressed group, with 34% of HCV patients suffering from depression compared with only 15% of IBS and 11% of IBD patients.

Moreover, HCV patients were considerably more affected by psychological disturbance than IBD patients on 6 subscales of the SCL90 and more impaired than IBS patients on one SCL90 subscale. Multiple regression showed that demographic characteristics such as age, education, and disease activity did not explain this difference, making it less likely to be due simply to a selection bias of the participants. Although, time since diagnosis might have partly explained this result on the SCL90 PST (Positive Symptom Total) subscale, as HCV participants with longer disease duration had higher a mean PST score than those with shorter disease duration. Moreover, the HCV group was more homogenous than the remaining groups in time since diagnosis as indicated by the narrower spread around the mean in this variable. The fact that IBD patients were the group least affected by psychological problems compared to IBS and HCV participants indicated that the overt physical morbidity of an inflammatory illness did not directly drive psychological morbidity in these patients.

Although, in our group, HCV patients experience more psychological problems than IBS and IBD participants, their prevalence of depression was comparable to the reported average (30%) for other chronic diseases [20], whereas in our IBS and IBD groups, the prevalence of depression was significantly lower than usually reported [4,21-23]. The prevalence of anxiety in all three groups (42%), on the other hand, was much elevated compared with the usually reported 20% prevalence in chronic diseases [24]. In comparison with other studies on prevalence of anxiety in HCV, IBD and IBS, the current prevalence seems elevated for HCV and IBD participants [23,25], however it is consistent with existing data for IBS [26].

The fact that 56% of IBS participants and 36% of IBD participants had active disease may contribute to this finding, as frequent visits to toilets and, in consequence, a disruption to normal functioning may lead to anxiety. In HCV, however, the cause is not as clear but may, in part be related to some or all of the following factors: disease stigma; frequent coexistent drug and alcohol addiction; and co-occurrence of HCV with other diseases such as AIDS [19]. In addition, the psychological status of HCV patients is also known to be dependant on whether they are aware of their HCV positivity [27], with those aware of their diagnosis being more impaired than those unaware, suggesting the influence of cognitive factors in addition to "medical" or disease and treatment related issues. Another factor contributing to the finding of high rates of anxiety and depression in HCV may be psychological side-effects of treatment with Interferon-Ribavirin [28]. In particular, Interferon is thought to affect pathways that are involved in the aetiology of depression [29]; however, the strength of the association between Interferon and depression is disputed as the available studies do not commonly use validated measures of depression [30].

Another hypothesis is that the actual virus may directly effect brain functions [31] and be capable of infecting the central nervous system causing neurological damage. It has been reported that this neurological damage may manifest itself through cognitive impairment in the domains of attention, concentration and information processing speed [32]. It is unclear whether this may also lead to depression or anxiety, however, this possibility should not be ignored.

Finally, it is important to acknowledge that the psychological screening questionnaires used in the present study only indicate the likelihood of anxiety and depression, and ideally, definite cases need to be formally diagnosed by a mental health specialist using a structured psychiatric interview based on the Diagnostic and Statistical Manual of Mental Disorders (DSM-IV) classification [12]. For this reason, the prevalence of psychological problems noted in this study should be interpreted with caution. Future studies would benefit from incorporating structured psychiatric interviews to confirm diagnoses.

## Conclusion

Based on our data, patients with HCV should be screened for depression, and all three groups for anxiety, as recognition and treatment of these psychological co-morbidities has the potential to improve patient outcomes. This could be done with the use of free and simple screening measures such as the Depression, Anxiety and Stress Scale (DASS). Unquestionably, given the high prevalence of anxiety we have reported, future studies should focus on interventions targeting anxiety which appears to be an unrecognised problem in patients with gastrointestinal and hepatologic disorders. The role of psychological co-morbidities, and treatment of them, on clinical outcomes deserves further study.

## Authors' contributions

The authors contributed equally to this work.

AMW contributed to the conception of this manuscript, designed it, collected data, performed analysis and interpreted data, and wrote the first and final drafts.

DT contributed to the conception of this manuscript, revised it critically and gave the final approval of the version to be published.

JA assisted with study design, recruitment, contributed to the conception of this manuscript, revised the manuscript critically and contributed to the final draft.

NM revised the manuscript critically and contributed to the final draft.

IW revised the manuscript critically and contributed to the final draft.

HH assisted with recruitment, revised the manuscript critically and contributed to the final draft.

DH assisted with recruitment, revised the manuscript critically and contributed to the final draft.

GH revised the manuscript critically and contributed to the final draft.

All authors read and approved the final manuscript.

## References

[B1] Mayer EA Craske M Naliboff BD Depression, anxiety, and the gastrointestinal system J Clin Psychiatry 2001 62 Suppl 8 28 36 discussion 37. 12108819

[B2] Kuchler T Bestmann B Rappat S Henne-Bruns D Wood-Dauphinee S Impact of psychotherapeutic support for patients with gastrointestinal cancer undergoing surgery: 10-year survival results of a randomized trial J Clin Oncol 2007 25 19 2702 2708 10.1200/JCO.2006.08.2883 17602075

[B3] Deter HC Keller W von Wietersheim J Jantschek G Duchmann R Zeitz M Psychological treatment may reduce the need for healthcare in patients with Crohn's disease Inflamm Bowel Dis 2007 17230495 10.1002/ibd.20068

[B4] Mittermaier C Dejaco C Waldhoer T Oefferlbauer-Ernst A Miehsler W Beier M Tillinger W Gangl A Moser G Impact of Depressive Mood on Relapse in Patients With Inflammatory Bowel Disease: A Prospective 18-Month Follow-Up Study Psychosom Med 2004 66 1 79 84 10.1097/01.PSY.0000106907.24881.F2 14747641

[B5] Fullwood A Drossman DA The relationship of psychiatric illness with gastrointestinal disease Annu Rev Med 1995 46 483 496 10.1146/annurev.med.46.1.483 7598481

[B6] Crone C Gabriel GM Comprehensive review of hepatitis C for psychiatrists: risks, screening, diagnosis, treatment, and interferon-based therapy complications J Psychiatr Pract 2003 9 2 93 110 10.1097/00131746-200303000-00002 15985921

[B7] Barratt HS Kalantzis C Polymeros D Forbes A Functional symptoms in inflammatory bowel disease and their potential influence in misclassification of clinical status Aliment Pharmacol Ther 2005 21 2 141 147 10.1111/j.1365-2036.2005.02314.x 15679763

[B8] Holtmann MH Galle PR Neurath MF Treatment of patients with Crohn's disease and concomitant chronic hepatitis C with a chimeric monoclonal antibody to TNF Am J Gastroenterol 2003 98 2 504 505 10.1111/j.1572-0241.2003.07245.x 12591081

[B9] Gwee KA Leong YL Graham C McKendrick MW Collins SM Walters SJ Underwood JE Read NW The role of psychological and biological factors in postinfective gut dysfunction Gut 1999 44 3 400 406 10026328 10.1136/gut.44.3.400 PMC1727402

[B10] Barbara G De Giorgio R Stanghellini V Cremon C Salvioli B Corinaldesi R New pathophysiological mechanisms in irritable bowel syndrome Aliment Pharmacol Ther 2004 20 Suppl 2 1 9 10.1111/j.1365-2036.2004.02036.x 15335408

[B11] Walker EA Roy-Byrne PP Katon WJ Li L Amos D Jiranek G Psychiatric illness and irritable bowel syndrome: a comparison with inflammatory bowel disease Am J Psychiatry 1990 147 12 1656 1661 2103735 10.1176/ajp.147.12.1656

[B12] Diagnostic and statistical manual of mental disorders [electronic resource] : DSM-IV-TR

[B13] Derogartis LR SCL-90-R Symptom Checklist-90-R. Administration, Scoring, and Procedures Manual 1994 Minneapolis: NCS Pearson

[B14] Ware JE Kosinski M Keller SD A 12-Item Short-Form Health Survey: construction of scales and preliminary tests of reliability and validity Med Care 1996 34 3 220 233 10.1097/00005650-199603000-00003 8628042

[B15] Best WR Becktel JM Singleton JW Kern F Jr Development of a Crohn's disease activity index. National Cooperative Crohn's Disease Study Gastroenterology 1976 70 3 439 444 1248701

[B16] Walmsley RS Ayres RC Pounder RE Allan RN A simple clinical colitis activity index Gut 1998 43 1 29 32 9771402 10.1136/gut.43.1.29 PMC1727189

[B17] WORLD MEDICAL ASSOCIATION DECLARATION OF HELSINKI: Ethical Principles for Medical Research Involving Human Subjects 1964 Helsinki: World Medical Association

[B18] Ladep NG Obindo TJ Audu MD Okeke EN Malu AO Depression in patients with irritable bowel syndrome in Jos, Nigeria World J Gastroenterol 2006 12 48 7844 7847 17203531 10.3748/wjg.v12.i48.7844 PMC4087553

[B19] Golden J Conroy RM O'Dwyer AM Golden D Hardouin JB Illness-related stigma, mood and adjustment to illness in persons with hepatitis C Soc Sci Med 2006 63 12 3188 3198 10.1016/j.socscimed.2006.08.005 17010490

[B20] Cavanaugh SV Clark DC Gibbons RD Diagnosing depression in the hospitalized medically ill Psychosomatics 1983 24 809 815 6647726 10.1016/S0033-3182(83)73151-8

[B21] Andrews H Barczak P Allan RN Psychiatric illness in patients with inflammatory bowel disease Gut 1987 28 12 1600 1604 3428687 10.1136/gut.28.12.1600 PMC1433950

[B22] Cole JA Rothman KJ Cabral HJ Zhang Y Farraye FA Migraine, fibromyalgia, and depression among people with IBS: a prevalence study BMC Gastroenterol 2006 6 26 17007634 10.1186/1471-230X-6-26 PMC1592499

[B23] Kraus MR Schafer A Csef H Scheurlen M Faller H Emotional state, coping styles, and somatic variables in patients with chronic hepatitis C Psychosomatics 2000 41 5 377 384 10.1176/appi.psy.41.5.377 11015623

[B24] Rodin G Craven J Littlefield C Depression in the Medically Ill. An Integrated Approach 1991 New York: Brunner/Mazel, Publishers

[B25] Simren M Axelsson J Gillberg R Abrahamsson H Svedlund J Bjornsson ES Quality of Life in Inflammatory Bowel Disease in Remission: The Impact of IBS-LIke Symptoms ans Associated Psychological Factors Am J Gastroenterol 2002 97 2 389 396 11866278 10.1111/j.1572-0241.2002.05475.x

[B26] Creed F Guthrie E Ratcliffe J Fernandes L Rigby C Tomenson B Read N Thompson DG Does psychological treatment help only those patients with severe irritable bowel syndrome who also have a concurrent psychiatric disorder? Aust N Z J Psychiatry 2005 39 9 807 815 10.1111/j.1440-1614.2005.01686.x 16168039

[B27] Rodger AJ Jolley D Thompson SC Lanigan A Crofts N The impact of diagnosis of hepatitis C virus on quality of life Hepatology 1999 30 5 1299 1301 10.1002/hep.510300504 10534353

[B28] De Bie J Robaeys G Buntinx F Hepatitis C, interferon alpha and psychiatric co-morbidity in intravenous drug users (IVDU) : guidelines for clinical practice Acta Gastroenterol Belg 2005 68 1 68 80 15832590

[B29] Loftis JM Hauser P The phenomenology and treatment of interferon-induced depression J Affect Disord 2004 82 2 175 190 10.1016/j.jad.2004.04.002 15488246

[B30] Dieperink E Willenbring M Ho SB Neuropsychiatric symptoms associated with hepatitis C and interferon alpha: A review Am J Psychiatry 2000 157 6 867 876 10.1176/appi.ajp.157.6.867 10831463

[B31] Forton DM Allsop JM Main J Foster GR Thomas HC Taylor-Robinson SD Evidence for a cerebral effect of the hepatitis C virus Lancet 2001 358 9275 38 39 10.1016/S0140-6736(00)05270-3 11454379

[B32] Forton DM Allsop JM Cox IJ Hamilton G Wesnes K Thomas HC Taylor-Robinson SD A review of cognitive impairment and cerebral metabolite abnormalities in patients with hepatitis C infection Aids 2005 19 Suppl 3 S53 63 10.1097/01.aids.0000192071.72948.77 16251829

[B33] Avery J Dal Grande E Taylor A Quality of life in South Australia as measured by the SF12 Health Status Questionnaire: Population norms for Trends from 1997 to 2003 Department of Human Services 2004

